# Accuracy of physical activity assessment during pregnancy: an observational study

**DOI:** 10.1186/1471-2393-11-86

**Published:** 2011-10-31

**Authors:** Katie M Smith, Randal C Foster, Christina G Campbell

**Affiliations:** 1 Department of Food Science and Human Nutrition, Iowa State University, 220 MacKay Hall, Ames, Iowa, 50011, USA

## Abstract

**Background:**

Prenatal physical activity may improve maternal and infant health and lower future disease risk for both mother and baby; however, very few physical activity assessment methods have been validated for use during pregnancy. The purpose of this study was to evaluate the accuracy of a subjective physical activity record (PAR) and an objective activity monitor, against a reference standard to quantify moderate and vigorous physical activity (MVPA) in pregnant women. The reference standard was based on participant interviews to determine if a woman was an exerciser and confirmed with information obtained from the PAR and a heart rate monitor.

**Methods:**

Fifty-two pregnant women completed a physical activity record (PAR) and wore a SenseWear^® ^Mini Armband (SWA) activity monitor over a 7-day period at 18 weeks gestation. Total minutes spent in MVPA were totaled from both modalities and evaluated against the reference standard using contingency analysis and Pearson's chi-square test to evaluate the number of women meeting minimum prenatal physical activity recommendations (at least 3, 30 minute sessions of exercise per week). Both modalities were also tested individually and collectively to assess their ability as indicators of activity using empirically determined cut-offs as indicated by receiver-operator characteristic curves. These experimentally-derived criteria were also tested with Pearson's chi-square test.

**Results:**

According to the reference standard, 13 of 52 participants (25%) met the criterion of 3, 30 minute sessions of volitional, moderate-intensity activity. When compared to the reference standard, both the PAR and SWA overestimated exercise status; 42 (81%) and 52 (100%) participants, respectively, achieved 90 minutes of MVPA (P < 0.0001 for both comparisons). Single-modality predictors of MVPA did not show a significant correlation. A composite predictor of MVPA offered the most favorable option for sensitivity and specificity (true positives, n = 8 and true negatives, n = 36) using cut-offs of 280 and 385 minutes/week for the PAR and SWA, respectively.

**Conclusion:**

Compared to the reference standard, time spent in MVPA obtained from the PAR or SWA overestimated the prevalence of women meeting prenatal exercise recommendations. The most accurate predictor of women meeting current prenatal exercise guidelines was identified by using the PAR and SWA collectively.

## Background

Physical activity during pregnancy has been shown to improve health outcomes for both the mother and the fetus. Maternal exercise may reduce the risk for certain pregnancy-related complications such as gestational diabetes [1-5]. In addition, some evidence suggests physical activity is associated with a shorter active labor and a reduction in back pain, insomnia, and anxiety [3,6,7]. Fetal outcomes improved by maternal physical activity include higher APGAR scores at birth [6] and increased fat-free mass at birth [8].

To date, there are three different guidelines for prenatal physical activity in the United States. While these recommendations may appear similar, subtle discrepancies exist in the language. The American College of Obstetrics and Gynecology (ACOG) currently recommends that pregnant women accumulate 30 minutes or more of moderate-intensity exercise on most, if not all, days of the week if no medical or obstetric complications are present [2]. Recommendations set forth by the U.S. Department of Health and Human Services (DHHS) in the 2008 Physical Activity Guidelines for Americans state that pregnant women should engage in a minimum of 150 minutes of moderate-intensity aerobic activity a week, even if not physically active prior to pregnancy [9]. The American College of Sports Medicine (ACSM) currently recommends a minimum of 3 exercise sessions completed in at least 15 minute sessions, gradually increasing to 30 minutes per day, preferably all days of the week [10]. Recommendations are similar in Canada [11], Denmark [12], Great Britain [13], Norway [14] and Australia [15].

To determine whether prenatal physical activity recommendations are being met, accurate methods must be used to quantify time spent in moderate-vigorous physical activity. Current subjective tools to quantify prenatal physical activity include logs (also referred to as records (PAR) or diaries), surveys, recalls and questionnaires such as the PPAQ [16] and the Modified Kaiser Physical Activity Survey [17]. Objective methods used to measure physical activity in other pregnancy-related studies have included accelerometer-based activity monitors such as the Actigraph [17,18] and RT3 [19] and pedometers such as the Yamax Digiwalker SW-200 [19,20], New Lifestyles NL 2000 [19], and Omron HJ-7201TC [19]. The most common pedometers are limited to reporting total step counts rather than intensity or duration of activity; however, they are relatively cheap and easy to use. Accelerometer-based activity monitors can detect acceleration in at least one axis of movement as well as the intensity and duration of activity or exercise bouts. The downside to these monitors generally includes the need to remove them when exposed to water (e.g. swimming and showering) and the increased cost of the devices in comparison to other methods such as surveys and pedometers. However, these monitors are less expensive and intrusive compared to other objective methods of measuring energy expenditure such as indirect calorimetry and doubly labeled water. Accelerometer-based activity monitors also require advanced algorithms to process and analyze the data. Despite these limitations, these monitors are currently the most accepted approach to assess physical activity in a free-living situation.

Receiver-operator characteristic curves (ROC) can be constructed to evaluate the accuracy of physical activity assessment methods. Using a reference standard, the ROC analysis provides information about each method evaluated to delineate which subjects are correctly identified as exercisers (true positives), correctly identified as non-exercisers (true negatives), and which subjects are misclassified (false positives and false negatives). This information obtained from the ROC allows the investigator to evaluate the sensitivity (ensuring the maximum number of actual exercisers identified as exercisers) and specificity (ensuring the maximum number of actual non-exercisers identified as non-exercisers) [21].

The purpose of this study was to evaluate the accuracy of two physical activity assessment methods, a subjective physical activity record (PAR) and an objective activity monitor, against a reference standard, to quantify moderate and vigorous physical activity (MVPA) in pregnant women. The reference standard was based on participant interviews to determine if a woman was an exerciser and confirmed with information obtained from the PAR and heart rate monitor data. A secondary aim was to optimize the sensitivity and specificity of the modalities evaluated to minimize false rates when assessing physical activity. It was hypothesized that the objective activity monitor would be a more accurate assessment method to identify women meeting current prenatal physical activity recommendations.

## Methods

### Participants

Sixty-nine pregnant women were recruited in and around Ames, Iowa, via a convenience sample as part of a larger observational study analyzing physical activity and omega-3 fatty acid intake in pregnant women living in a non-coastal community. Primary recruitment methods included mass emails to faculty, staff and students at Iowa State University, advertisements on campus and in the community, newspapers, and at local obstetric clinics. Ten women withdrew from the study or were excluded after enrollment due to time constraints, miscarriage, diagnosis of twins, or medical complications. In addition, seven women did not have complete data sets. Therefore, fifty-two women were included in this analysis.

Participants were recruited between May 2009 and May 2010. Non-smoking women between the ages of 18 and 45 with a singleton pregnancy were enrolled prior to their 18^th ^week of gestation. Women were excluded if they had a history of chronic disease or planned to deliver outside of Ames, Iowa, or other partnering hospitals. Each participant's medical provider confirmed all aforementioned qualification criteria prior to their patient's participation in the study. Since the study was strictly observational, no medical pre-screens for exercise were required to participate. All participants provided written informed consent prior to participation. The study was approved by the Iowa State University Institutional Review Board.

### Data Collection

All participants met with a staff member at week 18 of gestation to complete a medical history questionnaire and complete an interview assessing regular physical activity patterns since becoming pregnant. Each woman also received instructions on how to properly record her daily activity for two separate methods (described below) over the next 7-days. Following the 7-day data collection period, participants met with a staff member to return the PAR and activity monitor.

#### Physical activity record

A subjective PAR was completed by each participant to document all daily activity (24-hours per day for all 7 days), including sleep time and any activity that occurred between going to bed at night and waking the next day (i.e. trips to the restroom, to the kitchen, to children's bedrooms, etc.). Activities performed each day were listed chronologically with reference to start and end time of each event. A space for descriptions was included to discuss intensity and further details of each activity if necessary.

#### Activity monitor

The SenseWear^® ^Mini Armband (Model Name: MF) (SWA), (BodyMedia, Inc., Pittsburgh, Pennsylvania) was worn on the upper left arm for all 7 days, 24-hours per day, to estimate energy expenditure. This device is an objective monitor that uses a combination of a triaxial accelerometer, skin temperature sensor, galvanic skin response sensor and thermometers (measure heat flux) to detect movement and predict energy expenditure using a proprietary algorithm (version 2.2). For each participant, a SWA monitor was configured with height, body weight, age, gender, smoking status, and handedness per the manufacturer's instructions. Participants were instructed to remove the armband only during times of water submersion (showering, swimming, etc.).

#### Participant characteristics

Anthropometric and demographic data collected included age, pre-pregnancy weight, height, parity, due date, ethnicity, education level and total household income. Body weight was measured using a Sunbeam (2008 Sunbeam^® ^Products, Inc., Boca Raton, Florida) analog scale with participants not wearing shoes. Height and pre-pregnancy weight were self-reported in the medical history questionnaire and used as a descriptive characteristic only. Participants were asked to classify their ethnicity as American Indian or Alaska Native, African American, Caucasian, Asian, Hispanic, or other.

### Data Analysis

#### Activity monitor

Raw data from the SenseWear analysis system were imported into MATLAB (Version R2008a, Mathworks, Natick, MA). Total time spent in moderate (3-5.9 METs), and vigorous (6-9 METs) physical activity were extracted from the data files, formatted, and totaled. Time discrepancies in the data were adjusted so all records were reflective of 7, 24-hour periods to equate to 1 week's time (n = 10,080 minutes). Time gaps were filled using an algorithm that first detected off-body time in the data and then filled these time gaps with 1 MET (equivalent to rest) [22]. Off-body time was present in the data mostly for the purposes of light-intensity activity such as bathing or showering and did not substantially alter the data.

#### Physical activity record

Data from the 7-day subjective PAR was entered into a spreadsheet (Microsoft, Redmond, WA) by a single individual. Each activity documented in the PAR, including sleep time, was assigned a metabolic equivalent (MET) using the Compendium of Physical Activities (CPA) [22]. A new version of the CPA was recently published [23]. We reviewed the PAR entries and MET levels that had been assigned for the specific activities from the old CPA and assessed if any of these would have changed categories (i.e. light to moderate or moderate to light) if we had used the new CPA. Although some of the specific MET values differed, none of the MET values for the activities entered in the PAR changed categories of intensity. Therefore our results would not have differed if the new CPA had been used in our original analysis of the PAR (data not shown). Each PAR was then verified to ensure it totaled 10,080 minutes, the time equivalent to a 7-day period. Due to intra-individual inconsistencies in start/end time of recording PAR data on the first and last day of data monitoring, eight records fell short of 10,080 minutes. Time was filled for these records on day 8 with CPA MET values equivalent to the previous identical week day until 10,080 minutes were reached.

Data from the PAR was then further analyzed on a day-by-day basis using algorithms developed in Microsoft Office Excel 2007. These algorithms allowed us to partition each 24-hour period into various levels of activity equivalent to the activity categorizations previously stated for the SWA. Total daily time spent in MVPA (> 3.0 METs) in the PAR was computed and compared to total daily time spent in MVPA (> 3.0 METs) as recorded by the SWA.

#### Data entry training

Since a MET level for each activity in the PAR had to be assigned by a data entry individual, it was imperative that each data entry individual was consistent with MET assignments. Those responsible for this task had to complete a training test. The test records consisted of PARs from this cohort of women. Total daily minutes spent in MVPA entered by the data entry individual had to be within five percent of the total daily MVPA minutes of the original analysis conducted by one of the authors. Upon successfully meeting these criteria on five test records, each data entry individual was allowed to enter and analyze data.

#### Reference standard

To identify the exercisers in our study population, the reference standard used the criterion of at least 3, 30 minute volitional sessions of MVPA. Data indicating frequency, duration, and type of exercise since becoming pregnant were self-reported in the medical history questionnaire. Additionally, each participant was verbally interviewed in person at the beginning of week 18 of gestation by a member of the research staff regarding physical activity since becoming pregnant. Each woman was asked "Do you regularly engage in at least 3, 30 minute sessions of moderate exercise per week?" Depending on the participant's response, the interviewer may have probed to determine if the activity was volitional or incidental, e.g. "I went for a walk" or "I walked from my parked car into the store", respectively. If the woman met the criterion of at least 3, 30 minute volitional sessions per week, she was provided a heart rate (HR) monitor (Polar E600, Polar Electro Oy, Kempele, Finland) and instructed to wear it while exercising during the following 7-day monitoring period. Not all women were given a HR monitor because we only used this information as an additional method to objectively confirm exercise sessions listed in the PAR. Furthermore we did not solely rely on the HR monitor data due to interference/noise associated with use of the HR monitor. At the follow-up visit, data collected by the participant during the preceding week was reviewed. An ACSM Certified Health Fitness Specialist (HFS) utilized the PAR and HR monitor data to confirm that the participant did or did not meet the exercise criterion. Therefore, the number of exercisers used as the reference standard was identified by the ACSM HFS using data from the initial interviews, the PAR, and the HR monitors, if applicable. In summary, if the woman stated that she met the criterion in the initial verbal interview, and this was confirmed by the PAR and the HR monitor, she was considered an "exerciser". If the woman answered "no" to regularly participating in at least 3-30 minute moderate exercise sessions per week, but her PAR indicated she completed at least this amount of activity, she was still considered an "exerciser." This criterion was established based on the ACSM Guidelines for Exercise Testing and Prescription [10], has been used in other prenatal physical activity studies [24,25], and allowed us to identify as many exercisers as possible within our population.

### Statistical analysis

The reference standard was used to evaluate the ability of the PAR and the SWA to accurately differentiate between exercisers and non-exercisers. The number of exercisers and non-exercisers identified with both modalities (PAR and SWA) for two different guidelines, 90 minutes (mirroring the reference standard) and 150 minutes MVPA (per DHHS guidelines) were determined. Next, each participant's categorization for each modality was compared to the reference standard, and the results were compared against the binomial distribution for categorical data.

Minute-accumulations of MVPA from both the PAR and SWA were used independently to differentiate between exercisers and non-exercisers. The participant categorization was compared to the reference standard using receiver operator characteristic (ROC) curves plotting sensitivity against the specificity at each cut-off for each modality. Models were compared by computing the area under the curve (AUC) of the ROC using the trapezoidal method. The AUC was computed and treated as the Mann-Whitney U statistic. The phi coefficient (Φ) was determined for each cut-off and this Φ was evaluated for significance of fit using the chi-square test as well as standard rules for effect size [26-28].

Data from the PAR and the SWA were combined factorially to form a composite measure so that each cut-off from the SWA was compared to each cut-off from the PAR. To be classified as an exerciser, both modalities had to agree that the participant met the criterion. Each of the approximately 2,000,000 categorizations was compared to the reference standard and the results were used to generate sensitivity, specificity, and Φ values. The chi-square test was used to determine a significant fit between the composite measure and reference standard.

Algorithms used to categorize the data through the use of cut-offs as well as algorithms to evaluate sensitivity, specificity, AUC, and Φ were developed and implemented in MATLAB R2008A. For all tests, significance was set at P < 0.05.

## Results

Subject characteristics are stated in Table 1. Most of these women were well-educated (85% had at least a bachelor's degree, n = 44), were of higher socioeconomic status (54% with household income ≥ $50,000, n = 28), and were predominantly Caucasian (92%, n = 48). For 52% (n = 27), this was their first pregnancy.

**Table 1:** Descriptive characteristics of participants

Participant characteristic (n = 52)	Mean ± SD
Age at enrollment (yrs)	28.9 ± 4.0

Pre-pregnancy weight (kg)	68.6 ± 14.2

Height (cm)	168.7 ± 7.3

Pre-pregnancy BMI (kg/m^2^)	24.3 ± 4.5

Values are presented as mean ± SD. Body mass index is abbreviated as BMI.

Using the criterion of 3, 30 minute sessions of volitional, moderate-intensity exercise (reference standard), 13 of 52 participants were classified as "exercisers." The PAR and the SWA identified 42 and 52 participants, respectively, who met an accumulated 90-minute total of MVPA. Since 81% to 100% of participants were classified as exercisers according to the PAR and the SWA, respectively, we used the 150 min MVPA DHHS guideline as a cut-off to assess sensitivity and specificity for each modality. When this cut-off was applied, the PAR and the SWA determined that 36 and 49 participants, respectively, met this more rigorous prenatal recommendation for physical activity (compared to 90 minutes). Using the 150 minute cut-off, the PAR had a sensitivity (number of true exercisers identified by a modality divided by the number of actual exercisers per the reference standard) of 92% and a specificity (number of true non-exercisers identified by a modality divided by the actual number of non-exercisers per the reference standard) of 38% while the SWA had a sensitivity of 100% and a specificity of 7.7% (Table 2). The contingency correlation coefficient for the PAR and the SWA was Φ = 0.289 and Φ = 0.183, respectively. These fits were not significant according to Pearson's chi-square test (P = 0.228 and P = 0.786, respectively).

**Table 2:** Ability of PAR and SWA to identify exercisers and non-exercisers against a reference standard

Test Outcomes	Reference Exercise Status	
	
	Exerciser	Non-exerciser
PAR exerciser	12 (True +)	24 (False +)

PAR non-exerciser	1 (False -)	15 (True -)

SWA exerciser	13 (True +)	36 (False +)

SWA non-exerciser	0 (False -)	3 (True -)

Test outcomes based on a cut-off of more than 150 minutes of moderate-to-vigorous physical activity per week compared against the reference standard for exerciser vs. non-exerciser. (PAR = physical activity record; SWA = SenseWear^® ^armband; + = positive; - = negative).

**Figure 1:**
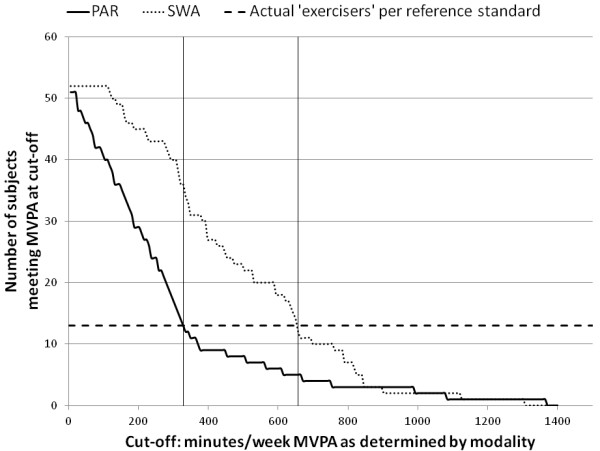
**Participants identified as meeting physical activity recommendations by a subjective and an objective assessment**. Participants meeting moderate-to-vigorous activity (MVPA) using two different modalities (PAR = physical activity record, SWA = SenseWear armband) in minutes per week, compared to actual number of participants meeting the reference standard (3, 30 minute volitional sessions per week). The horizontal line demonstrates the cut-off of MVPA minutes indicated by each modality required to classify the same number of exercisers identified by the reference standard. Participants identified at these cut-offs were not necessarily the same individual participants identified by the reference standard.

**Figure 2:**
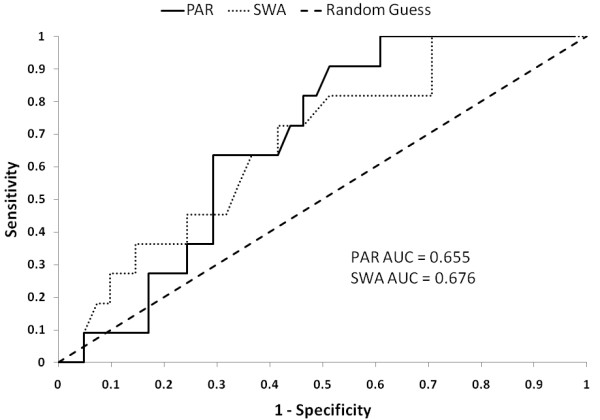
**Receiver operator characteristic curves for classifying activity in pregnant women**. Assessing moderate-to-vigorous physical activity (MVPA) using receiver operator characteristic (ROC) curves for two modalities (PAR = physical activity record, SWA = SenseWear armband). Sensitivity and specificity were determined through the entire range of cut-off times (1-1400 minutes) by comparing classification as an exerciser/non-exerciser determined by the modalities against the reference standard. "Random guess" is the line that would be obtained if classifications were made at random. AUC = area under the curve; 0.500 the AUC of a random guess.

Next, total MVPA minutes per day from the PAR and SWA were used to identify the optimum cut-off to improve the accuracy of each modality instead of using a predetermined cut-off (e.g. 150 minutes). To accomplish this, activity data from the PAR and SWA were analytically subjected to a range of time (1-1400 minutes) to categorize participants as exercisers and non-exercisers and these classifications were compared to the reference standard (Figure 1). Figure 1 demonstrates that the cut-off needed for either modality to identify 13 exercisers (per the reference standard) is much greater than the 150 minute cut-off; furthermore the actual subjects identified are different for each modality. Figure 1 also shows that the PAR is more specific and less sensitive at any given point throughout the range (1-1400 minutes), although the ROC in Figure 2 demonstrates that in terms of AUC, the models are almost identical (SWA AUC = 0.676, PAR AUC = 0.655). According to the results of the chi-square test, neither modality shows a significant correlation even when the cut-off is selected to optimize the sensitivity and specificity (PAR: 119 minutes or SWA: 448 minutes) (Table 3). This finding demonstrates that neither assessment method, standing alone, can acceptably identify exercisers and non-exercisers.

**Table 3:** Single-modality predictors of moderate-vigorous physical activity

Model		True		False			P-
		
Modality	Cut-off	Positive	Negative	Positive	Negative	Φ	value
PAR	119	13	13	26	0	0.333	0.124

PAR	105	13	12	27	0	0.316	0.158

PAR	112	13	12	27	0	0.316	0.158

SWA	448	10	24	15	3	0.333	0.124

SWA	294	13	12	27	0	0.316	0.158

SWA	301	13	12	27	0	0.316	0.158

Model shows the modality (PAR = physical activity record, SWA = SenseWear^® ^armband) and the cut-off, in minutes per week of recorded moderate-to-vigorous physical activity. True/false/positive/negative data shows the number of participants in each category (out of a total of 52 participants) compared to the reference standard. Φ is the 2 × 2 contingency correlation coefficient. Significance was set at P < 0.05.

Since neither modality alone performed adequately, data from the PAR and SWA were combined into a composite measure using a logic-based categorical system to better differentiate participants who engaged in recommended levels of MVPA. This composite was formed by classifying a participant as an exerciser if and only if both the PAR and the SWA agreed that the participant was an exerciser at a given cut-off. Models in best agreement with the reference standard are shown in Table 4. For example, using a cut-off of 280 minutes according to the PAR in conjunction with a cut-off of 385 minutes from the SWA showed a strong agreement (Φ = 0.571, P < 0.001) with the reference standard, with 8 correctly-identified exercisers and 36 correctly identified non-exercisers. This model had only 8 misclassified participants, with 3 non-exercisers identified as exercisers (false positives) and 5 exercisers identified as non-exercisers (false negatives). With a false-positive rate of < 8%, this model shows a strong ability to identify most non-exercisers. Using a lower cut-off value for the PAR (168 minutes) while keeping the SWA cut-off constant (385 minutes) gives a greater ability to detect exercisers (3 exercisers identified as non-exercisers; false negatives) at the expense of a slightly higher number of non-exercisers identified as exercisers (8 false positives), with a false-negative rate < 23% (Φ = 0.513, P < 0.005).

**Table 4:** Composite predictors of moderate-vigorous physical activity

Cut-off		True		False		Φ	P-value
		
PAR (min)	SWA (min)	Positive	Negative	Positive	Negative		
280	385	8	36	3	5	0.571	0.0007

182	385	10	32	7	3	0.544	0.0015

259	385	8	35	4	5	0.527	0.0024

280	350	8	35	4	5	0.527	0.0024

210	385	9	33	6	4	0.515	0.0032

168	385	10	31	8	3	0.513	0.0033

280	400	7	36	3	6	0.507	0.0039

259	350	8	34	5	5	0.487	0.0063

Cut-off shows the cut-off time for each modality (PAR = physical activity record, SWA = SenseWear^® ^armband) in minutes per week. True/false/positive/negative data shows the number of participants in each category (out of a total of 52 participants) compared to the reference standard. Φ is the 2 × 2 contingency correlation coefficient. Significance was set at P < 0.05.

## Discussion

The current study evaluated the accuracy of two physical activity assessment methods, a subjective PAR and an objective activity monitor, against a reference standard, to quantify MVPA in pregnant women. Per the reference standard, 25% of the participants were classified as exercisers. According to data from the PAR or the SWA, it appears that 81% and 100% of women in this study met our criterion to be classified as an exerciser (3 times per week, 30 minutes or more), respectively. Thus contrary to our hypothesis, the objective monitor was not a more accurate assessment method to identify women meeting current prenatal physical activity recommendations. Our study demonstrates that meeting current recommendations may be overestimated depending on the assessment tool used or the application of the data.

The importance of the ability to discern between active and non-active states cannot be understated. A system that falsely reports a high number of participants meeting a certain physical activity level (false positives) would lead to the conclusion that a high percentage of the population under study is meeting recommendations. On the other hand, a system that falsely reports a high number of participants not achieving a certain physical activity level (false negatives) would indicate that the majority of the population is not meeting recommendations. These inaccuracies would have broad implications on policy decisions as well as increase the difficulty of identifying participants in need of an intervention (if false positive rate is high) or filtering out participants not in need of an intervention (if false negative rate is high). Finally, these inaccuracies make it difficult to evaluate the impact of an intervention. Thus a secondary aim of this study was to identify the optimal sensitivity and specificity of the modalities evaluated to minimize false rates when assessing physical activity.

The criterion, at least 3, 30 minute sessions of volitional, moderate-vigorous intensity exercise was used as the reference standard because it allowed us to identify as many exercisers as possible and adhere to minimum prenatal physical activity recommendations. Even using this less rigorous definition to identify exercisers, only 13 of 52 women were classified as exercisers.

Other studies using both subjective [18,29,30] and objective [30,31] assessment methods support a similar low prevalence of women meeting current prenatal physical activity recommendations as identified by our reference standard. Results from the Center for Disease Control's (CDC) Behavioral Risk Factor Surveillance System (BRFSS) data in 2000 revealed 16% of pregnant women completed at least 3, 20 minute bouts of vigorous leisure activity (or 5, 30 minute bouts of moderate leisure activity) [29]. Similarly, 23% of pregnant women surveyed by the 1999-2006 National Health and Nutrition Examination Survey (NHANES) met moderate-vigorous activity DHHS recommendations [18]. Studies using objective assessment methods identified the same prevalence with 11-14% of women achieving physical activity recommendations when assessed with a pedometer [30] and an accelerometer [31]. Conversely, other studies [32-34] report prevalence much higher than reported by the previously mentioned studies and our reference standard. A retrospective survey administered 6-32 months postpartum reported approximately 50% of women exercised during pregnancy [32] and the Avon Longitudinal Study of Parents and Children had nearly 50% of women report at least 3 hours of strenuous activity (defined as activity that induced sweating) during the 18^th ^week of gestation [33]. Additionally, McParlin et al. [34] identified 62-71% of pregnant women achieving at least 30 minutes of MVPA per day at 13, 26 and 36 weeks of gestation according to *accumulated *MVPA occurring throughout the day (as reported by an accelerometer). Results from the PAR and SWA in the current study support the findings of McParlin et al. and demonstrate that accumulated MVPA reveals exercise prevalence far higher than indicated by our reference standard, calling into question the interpretation of the data collected from these tools.

One potential source of error in the amount of MVPA reported in any study assessing physical activity during pregnancy using the CPA, may be due to the use of the MET to quantify physical activity [35,36]. Although the CPA was not developed to be used for adults with metabolically altered conditions [22], it is currently not well established to what extent the MET values may differ during pregnancy. Chasan-Taber et al. [37] found conflicting differences in MET values for four household tasks (window washing, dusting, vacuuming, and laundry). Mean MET values differed from the respective CPA values by as much as 43% higher than the CPA MET for laundry and 23% lower than the CPA MET for vacuuming. The MET is a ratio of energy expenditure during an activity to energy expenditure at rest and is dependent upon resting metabolic rate (RMR) for each individual. The basal metabolic rate (BMR) increases as a result of pregnancy [38,39]. If the MET overestimates energy expenditure during pregnancy, this may provide partial explanation for the large majority of participants identified as having achieved our exercise criterion as indicated by the PAR in the current study.

The version of the SWA used in the current study has previously been validated against doubly-labeled water in non-pregnant adults (r^2 ^= 0.71, P < 0.001; ICC = 0.85 (95% CI = 0.92--0.76)) [40]. Additionally, the study reported this version of the armband (SenseWear^® ^Mini, Model Name: MF) to be more accurate than a previous version (Pro_3_) when tested in non-pregnant populations [40]. The ability of the SWA to predict energy expenditure in early pregnancy has also been evaluated against indirect calorimetry [r^2 ^= 0.678; Smith et al. 2011, unpublished results]. However, there were limitations in the ability of the monitor to discern between flat and inclined walking and produced large variability in results for low-intensity activities (e.g. sweeping and folding laundry). Since a large portion of energy expenditure in pregnant women has been reported from low-intensity activities [34,41,42], this may explain some of the overestimation of MVPA by the SWA in the current study.

An additional explanation for the overestimation of MVPA reported in the SWA could be due to the fact that the SWA *accumulates *time for both incidental and volitional activities. Thus, the SWA is very capable of detecting MVPA, yet cannot differentiate between volitional or incidental activity. When the SWA is downloaded into the company's software program, *accumulated *minutes spent in sedentary, moderate and vigorous activity are reported. Some current guidelines [9,10] suggest physical activity be performed in minimum bouts, such as at least 10 minutes, thus using accumulated physical activity per day without regard to bouts, may result in an overestimation of women meeting current physical activity guidelines. This is a probable explanation as to why the objective monitor did not perform as well as hypothesized. Additional programming of the SWA data beyond the manufacturer's algorithms is needed to identify a minimum bout of activity thereby potentially improving the accuracy of the device as a stand-alone assessment tool.

There are some limitations to this study. The use of a non-waterproof sensor that requires users to remove the device when submerged in water could result in an underestimation of physical activity by the SWA if off-body time includes a substantial amount of MVPA such as swimming. Physical activity records of seven women indicated swimming during the week of data collection, however, only one of these women was considered an exerciser by the reference standard and none of the other six women reported more than one session of swimming during the week. Off-body time activities were identified from the PAR; however, to assess these two modalities independently, the activity from the PAR was not assigned a CPA MET to fill non-wear time of the SWA. Additionally, the SWA showed that all 52 women met the criterion without correcting for activity-specific off-body time. Thus, the use of the 1-MET substitution for off-body time did not alter our results since the SWA already identified the maximum number of exercisers possible.

## Conclusions

Compared to our reference standard, time spent in MVPA obtained from the PAR or the SWA overestimated the prevalence of women meeting prenatal exercise recommendations. The PAR and SWA used as a composite predictor of MVPA provided the optimal sensitivity and specificity to identify pregnant women meeting current physical activity guidelines. Data from the PAR or SWA requires additional programming to extrapolate bouts of MVPA to improve the accuracy of either modality as a stand-alone assessment tool. Analysis of physical activity data to determine the prevalence of individuals meeting current guidelines should take into consideration the assessment of accumulated, incidental activity versus volitional bouts of activity. Further research is needed to evaluate the effect of accumulated activity or intentional exercise performed in minimum bouts on maternal and fetal health outcomes.

## Competing interests

The authors declare that they have no competing interests.

## Authors' contributions

KS chaired recruitment of participants, collected and analyzed the data, provided the expert assessment on physical activity quantification, and drafted and revised the manuscript. RF carried out the statistical analyses, and assisted in drafting and revising the manuscript. CC designed the study, served as the primary investigator, and assisted in drafting and revising the manuscript. All authors read and approved the final manuscript.

## Pre-publication history

The pre-publication history for this paper can be accessed here:

http://www.biomedcentral.com/1471-2393/11/86/prepub
